# The association between body mass index and mortality among Asian peritoneal dialysis patients: A meta-analysis

**DOI:** 10.1371/journal.pone.0172369

**Published:** 2017-02-16

**Authors:** Jing Liu, Xiaoxi Zeng, Hyokyoung G. Hong, Yi Li, Ping Fu

**Affiliations:** 1 Division of Nephrology, Kidney Research Institution, West China Hospital of Sichuan University, Chengdu, Sichuan, China; 2 West China Biomedical Big Data Center, West China Hospital of Sichuan University, Chengdu, Sichuan, China; 3 Department of Statistics and Probability, Michigan State University, East Lansing, Michigan, United States of America; 4 Department of Biostatistics, University of Michigan, Ann Arbor, Michigan, United States of America; The University of Tokyo, JAPAN

## Abstract

**Background:**

Previous studies have revealed that increased body mass index (BMI) is associated with decreased mortality among hemodialysis patients. However, few studies have dealt with the association between BMI and mortality among patients undergoing peritoneal dialysis (PD) and even fewer studies have focused on the Asian PD patients. The reported studies were often non-conclusive and some even yielded contradictory results. This paper, to our best knowledge, registers the first attempt to systematically review the current literature and summarize new results on the association between BMI and mortality among the Asian PD population.

**Method:**

A systematic literature review was performed in Medline and EMBASE to identify relevant cohort studies on all-cause and cardiovascular disease (CVD) mortality stratified by BMI categories tailored to Asians among the Asian PD population. We meta-analyzed individual results based on a random effect model, strictly complying with Preferred Reporting Items for Systematic Reviews and Meta-analysis.

**Results:**

The paper reviews seven cohort studies with a total of 3,610 Asian PD patients. Obese group (BMI = 25–29.9 kg/m^2^) was associated with higher risk of all-cause mortality (HR = 1.46, 95%CI [1.07–1.98]; p = 0.02) and CVD mortality (HR = 2.01, 95%CI [1.14–3.54]; p = 0.02), compared to the normal group (BMI = 18.5–22.9 kg/m^2^). The underweight group (BMI<18.5kg/m^2^) was also associated with an elevated risk of all-cause mortality (HR = 2.11, 95%CI [1.46–3.07]; p<0.001). No significant associations between BMI with all-cause mortality were found among the overweight group (23–24.9 kg/m^2^) (HR = 1.00, 95%CI [0.76–1.32]; p = 0.9). The association between BMI and CVD mortality risk among the underweight and overweight groups was found nonsignificant (p = 0.5 and 0.6 respectively).

**Conclusion:**

Obesity is associated with increased mortality in Asian PD patients. The study indicates a “V-shaped” trend in the association between BMI and mortality in these patients.

## Introduction

Among the general population, increased Body Mass Index (calculated as weight in kilograms divided by the square of the height in meters), has been shown to be associated with increased all-cause mortality and cardiovascular disease (CVD) mortality [1–3]. Especially, the relationship between Body mass index (BMI) and all-cause mortality risk is “J- or V-shaped”, which has been well-documented in literature [4–6].

Conflicting results, though, have been reported among the peritoneal dialysis (PD) patients. A recent meta-analysis showed that higher BMI was associated with lower mortality among the PD patients [7], however some studies have disagreed[8–10]. It is also not known whether these results apply to the Asian PD patients, as the association between BMI and mortality risk among the PD patients was reported to vary among various ethnic groups [11, 12]. This paper presents the first systematic review of work on detecting effects of BMI on mortality among the Asian PD patients, and conducts a meta-analysis to quantitatively assess the impact of BMI on all-cause and CVD mortality among this unique population. In general, the mean or median BMI for the Asian population is lower than that for non-Asian populations. Hence the BMI distribution of Asians is shifted to the left, compared that of non-Asians. Thus direct applications of the current World Health Organization (WHO) BMI cut-offs to the Asian population may lead to the underestimation of obesity-related risks among the Asian populations. In this study we perform meta-analysis with a modified BMI categorization for Asians as recently recommended by WHO[13], namely, underweight (< 18.5kg/m^2)^, normal (18.5–22.9 kg/m^2^), overweight (23–24.9 kg/m^2^), obese I (25–29.9 kg/m^2^) and obese II (≥ 30 kg/m^2^).

## Method

This meta-analysis was performed complying with Preferred Reporting Items for Systematic Reviews and Meta-analysis (PRISMA, shown in **S1 Table**) [14], a rigorous and prevailing guideline for meta-analysis writing. No protocol exists for this meta-analysis. Neither ethics board review nor informed consent was required due to no participation of human subjects involved.

### Information source and search strategy

To identify eligible studies on the association between BMI and mortality in PD patients, two reviewers (JL and XXZ) searched PubMed (MEDLINE) and EMBASE for articles published in English from 1974 week 1 to 2016 week 19. The key words “peritoneal dialysis”, “continuous ambulatory peritoneal dialysis (CAPD)”, “body mass (index)”, “overweight”, “obesity”, combined with “mortality”, “mortality risk”, “survival” and combinations of these were used. References cited in the retrieved articles were also examined to find relevant studies that had not been identified by database searches. The final inclusion of articles was determined by consensus between two senior co-authors. **S1 File** shows the detail of the search strategy.

### Selection criteria

Studies were screened by three reviewers (JL, XXZ and PF) independently. Inclusion criteria included: (i) cohort studies; (ii) patients diagnosed with end-stage renal disease (ESRD) undergoing PD; (iii) articles that reported the association between BMI and mortality in PD patients in terms of hazard ratio (HR) or relative risk (RR) accompanied with 95% confidence interval (CI) or sufficient information to calculate these outcomes. We excluded: (i) abstracts, letters, editorials, expert opinions, case reports, and reviews; (ii) studies that included only non-Asian PD patients; and (iii) studies in which patients received renal replacement therapy other than PD.

Disagreement was solved by discussion with another reviewer (PF).

### Data collection

Data were extracted by two independent reviewers (JL and XXZ) using standardized forms. Data recorded included the first authors’ names, years of publication, the locations of studies, means of patients’ ages at enrollment, study design, numbers of total patients, medians of follow-up, BMI categorization criteria, and adjusted multivariable of hazard ratios (HRs) or relative risks (RRs) accompanied with 95% CIs for different categories of BMI.

### Quality assessment

Two reviewers (JL and XXZ) assessed the quality of studies using Newcastle-Ottawa Quality Assessment Scale (NOS) for cohort studies[15]. The NOS scale scores the quality of three domains separately: selection (a maximum of 4 points), comparability (a maximum of 2 points) and outcomes (a maximum of 3 points). Since no unified standards are well established, we defined the studies as ‘‘Good” (7–9 points), ‘‘fair” (4–6 points) or ‘‘poor” (0–3 points) using the total score. We included studies with an at least fair rating to maintain quality control.

### Statistical analysis

Stata V.13 (Stata Corporation, College Station, Texas, USA) was used for the meta-analysis, and p-values less than 0.05 were considered statistically significant, unless specified otherwise. The adjusted HR with a corresponding 95% CI was used as the effect size for all studies and the relative risks (RRs) were considered to be equal to HRs. This approach has been widely used in systematic review and/or meta-analysis[16–18]. Homogeneity across studies was tested by the Q statistic and quantitatively evaluated using the I^2^ statistic. For the Q statistic, a significant heterogeneity was defined by a p value<0.10. The values of I^2^ below 30% is defined as unimportant, 30–50% as moderate, 50–75% as substantial and >75% as considerable heterogeneity [19, 20]. We pooled HRs at the level of underweight, overweight, and obesity to assess the association between the BMI level and risk of mortality with the DerSimonian and Liard random effect model[21, 22]. This approach allows for variations of within-study as well as between-studies. Studies providing results stratified by White BMI categories[23] were converted according to the Asian BMI categorizations. The study presenting the single HR based on the continuous BMI[24] was treated as a separate report and was not used in the meta-analysis.

A priori meta-analysis was then performed in underweight, overweight and obese BMI groups to assess whether the conclusions were sensitive to restricting studies to subgroups based on: i) study designs (prospective vs. retrospective cohort study), ii) geographic areas (China vs. non-China region), and iii) NOS scores ranks (“good” vs. “fair”). Sensitivity analyses were performed by estimating a pooled estimate in the absence of an individual study and by identifying sources of significant heterogeneities.

### Publication bias

Since only 7 studies were qualified for our analysis, not satisfying the condition to apply funnel plot that the number of studies should be at least ten[25]. Thus the funnel plot accompanied with Egger’s regression asymmetry test and the Begg’s adjusted rank correlation test were not performed.

## Results

### Search results

**Fig 1:**
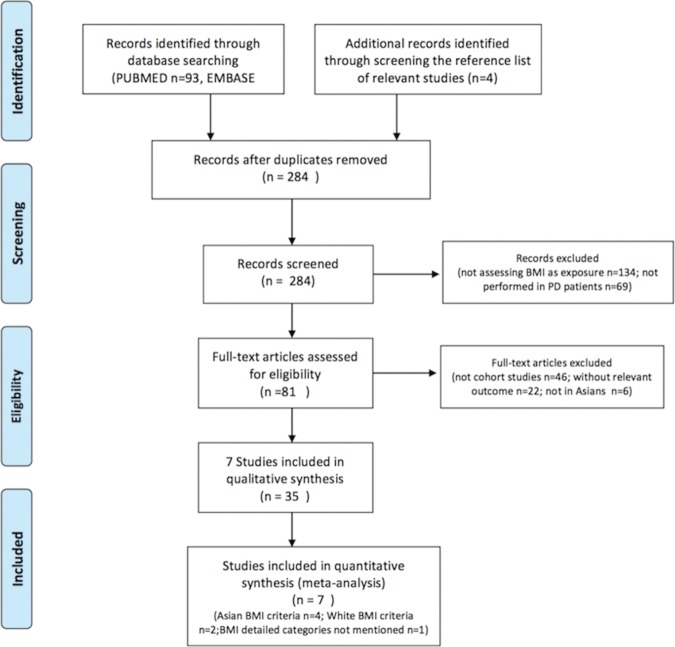
PRISMA Flow Diagram of Included Studies.

A total of 284 unique studies were identified from database search. Of 284 articles, 134 articles did not assess the mortality risk using BMI as predictor, and 69 articles were not performed in PD patients. We further excluded 46 reports which were not cohort studies, 22 reports with no relevant outcomes, and 6 studies with no Asian patients. Finally, 7 studies were included. Fig 1 shows the PRISMA flow diagram of screened, excluded, and included studies in the present meta-analysis [26].

### Study characteristics

The study characteristics of the seven included cohort studies[24, 27–32] with a total of 3,610 PD patients are shown in Table 1. There were three[27, 29, 30] prospective studies and four[24, 28, 31, 32] retrospective studies. Of them, four were conducted in China[28, 29, 31, 32], one in Korea[27], one in India[30], and one in the Asian part of Turkey[24].

**Table 1:** Characteristics and Results of Included Studies.

Study (author, year)	Country	Ethnicity	Age (Mean^a^)	Design /settings (PD modality)	Data Source	Sample Size	BMI Cutting Points / Mean^b^	Duration (y)	Mortality Outcome; P (Adjustments)	NOS ^c^ Score (Rank)
**Lam,M.F.2006[29]**	China	Chinese	52.45 ± 12.24	Prospective (CAPD)	Multiple Centers	294	18.5, 25, 30 /22.36±3.26	3.2	^$^**HR**_**3**_ 1.17(1.03,1.33);0.016	7 (good)
									(sex, age, CVD, DM, peritoneal Kt/V, peritoneal CrCl, GFR, urine volume, serum albumin)	
**Zhou, H. 2011[32]**	China	Chinese	51.8 ± 15.1	retrospective	Single Center	159	18.5,25,30 /23.5±NG	5	^$^**HR**_**3**_ 2.71(1.49,4.93);0.001	5 (fair)
									(age, DM, coronary vascular disease, congestive heart failure, Lp(a))	
**Unal, A. 2013[24]**	Turkey (Kayseri)	Turkish	51.0 ± 13.9	retrospective	Single Center	392	NG /23.6±4.2	3.7	^$^**HR** 1.05(1.01,1.09);0.01	5 (fair)
									(age, gender, BUN, Scr, Kt/V_crea_, blood lipid, albumin, blood pressure)	
**Kim, Y. K. 2014[27]**	Korea	Korea	56 ± 12	prospective	Multiple Centers	900	21.4,23.5,25.4 /23.6±3.2	2	^$^**HR**_**1**_ 3.00(1.26,7.15);0.01	8 (good)
									^$^**HR**_**2**_ 1.11(0.43,2.85);0.83	
									^$^**HR**_**3**_ 1.64(0.66,4.06);0.28	
									(age, gender, DM, Davis comorbidity score)	
**Kiran,V.R 2014[28]**	China	Chinese	63.4 ± 14.6	Retrospective (home PD)	Single Center	274	18.5,23,25 /21.97±3.23	3.6	^$^**HR**_**1**_ 1.91(1.07,3.40);0.028	7 (good)
									^$^**HR**_**2**_ 0.92(0.58,1.48);0.74	
									^$^**HR**_**3**_ 1.80(1.01,3.21);0.048	
									(age, CVD and DM)	
**Prasad.N 2014[30]**	India	Indian	52.6 ± 12.6	Prospective (CAPD)	Single Center	328	18.5,23,25 /NG	1.83	^$^**HR**_**1**_ 2.0(1.1,3.6);0.02	7 (good)
									^$^**HR**_**2**_ 1.1(0.6,1.9);0.88	
									^$^**HR**_**3**_ 0.8(0.4,1.6);0.56	
									(age, SGA, comorbidities, albumin, DM and rGFR)	
Xiong,L 2015**[31]**	China	Chinese	47.8 ± 15.0	Retrospective (CAPD)	Single Center	1263	18.5,23,25 /21.58±3.13	2.1	^$^**HR**_**1**_ 0.61(0.30,1.25);0.008	8 (good)
									^$^**HR**_**2**_ 0.99(0.61,1.59);0.82	
									^$^**HR**_**3**_ 1.54(0.94,2.52);0.60_)_	
									^#^**HR**_**1**_ 0.79(0.35,1.81);0.026	
									^#^**HR**_**2**_ 0.78(0.41,1,49);0.48	
									^#^**HR**_**3**_ 2.01(1.14,3.54);0.64	
									(age, sex, DM, CVD, MAP, hemoglobin, albumin, TC, TG, hs-CRP, rGFR, Kt/V_crea_	

**NG:** not given; **HR**_**1**_ = adjusted hazard ratio of PD patients’ mortality for underweight compared to normal BMI group; **HR**_**2**_ = adjusted hazard ratio of PD patients’ mortality for overweight compared to normal BMI group; **HR**_**3**_ = adjusted hazard ratio of PD patients’ mortality for obese compared to normal BMI group; **HR** = adjusted hazard ratio of PD patients’ mortality; **CVD** = cardiovascular disease; **DM** = diabetes mellitus; **CrCl** = creatinine clearance; **Lp(a)** = lipoprotein (a); **SGA** = subjective global assessment (a validated estimate of nutrition); **rGFR** = residual glomerular filtration rate; **MAP** = mean arterial pressure; **TC** = total cholesterol; **TG** = triglyceride; **hs-CRP** = high sensitive C-reactive protein; **Kt/V**_**crea**_ = urea clearance (Kt) normalized to total body water; **BUN** = blood urea nitrogen; **Scr** = serum creatinine.

^a, b^ Mean ± standard deviation

^c^ Newcastle-Ottawa Quality Assessment Scale.

^$^ all-cause mortality

^#^ cardiovascular disease mortality

The number of patients varied from 159[32] to 1,263[31]. All of the seven studies reported all-cause mortality risk outcome of the PD patients and only one study[31] provided CVD mortality risk outcome. While six studies used White BMI categories[29, 32] or recommended for Asian BMI[27, 28, 30, 31] population, one study[24] used the raw (continuous) BMI. Several potential confounders, such as age, gender, comorbidities (Diabetes Mellitus, CVD etc.), serum albumin, residual glomerular filtration rate (rGFR), and even high sensitive C-reaction protein (hs-CRP) and urea clearance (K_t_) normalized to total body water (K_t_/ V_urea)_ were taken into account. As for the quality assessment, five[27–31] studies received “good” (ranging from 7 to 8 scores) NOS ratings and two[24, 32] received a score of 5 corresponding to a “fair” NOS rating. Details of NOS scores distribution were shown in **S2 Table**.

### Primary result: Underweight, overweight, and obese vs. normal BMI analysis

**Fig 2:**
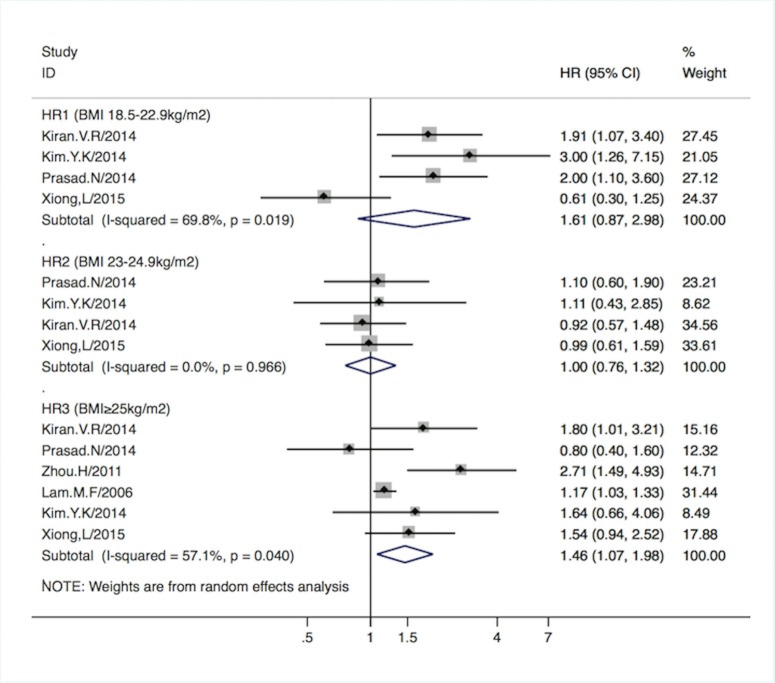
Pooled HR_1-3_ with 95%CI of all-cause mortality in different BMI groups. Solid diamond -HR from individual study; Shadowed square behind solid diamond- statistical weight that each study contributes to the random-effect summary estimate; Hollow diamond-the overall summary HR. Horizontal line-the study specific 95%CI. HR_1-3_ -adjusted hazard risk for underweight, overweight and obese group vs. normal BMI group based on Asian BMI categorization.

HR_1_, HR_2_, and HR_3,_ denoted the adjusted hazard ratio of all-cause mortality for underweight (BMI<18.5 kg/m^2^), overweight (BMI = 23–24.9 kg/m^2^), and obese groups (BMI = 25–29.9 kg/m^2^) compared to the normal weight group (BMI = 18.5–22.9 kg/m^2^). Asian PD patients in the obese group had a 46% higher all-cause mortality (HR_3_ 1.46, 95%CI [1.07–1.98]; p = 0.02), while the underweight (HR_1_ 1.61, 95%CI [0.87–2.98]; p = 0.1) and overweight (HR_2_ 1.00, 95%CI [0.76–1.32]; p = 0.9) groups did not present statistically different mortality, compared to the normal BMI group. One study[24] reported the HR (1.05, 95%CI [1.01–1.09]; p = 0.01) based on the continuous BMI scale, indicating higher BMI, was associated with higher all-cause mortality risk. (Forrest plots are shown in Fig 2.)

Xiong et al[31] reported CVD mortality risk in which the definition of the CVD mortality was death due to congestive heart failure, ischemic heart disease, cerebrovascular disease or sudden death (unnatural death within one hour from the occurrence of symptoms or lack of fatal indicators) according to standard clinical guideline [33, 34]. In this study, CVD deaths accounted for 59.1% of the total deaths during a follow-up of 6.8 years. The Asian PD patients in the obese group encountered higher risk of CVD mortality compared with normal BMI group (HR 2.01, 95%CI [1.14–3.54]; p = 0.02), while the risk for underweight and overweight groups were not statistically significant (p = 0.5 and 0.6 respectively).

### Subgroup analyses

**Fig 3:**
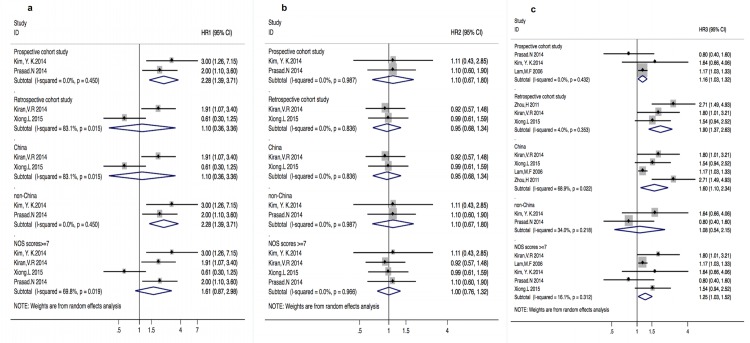
**a-c HRs with 95%CI of all-cause mortality in underweight, overweight and obese groups in different subgroups**. ***Fig 3a*-**underweight group (HR_1_); ***Fig 3b*-**overweight group (HR_2_); ***Fig 3c*-**obese group (HR_3_); Solid diamond -HR from individual study; Shadowed square behind solid diamond- statistical weight that each study contributes to the random-effect summary estimate; Hollow diamond-the overall summary HR. Horizontal line -the study specific 95%CI; HR_1_-adjusted hazard risk for underweight vs. normal group; HR_2_-adjusted hazard risk for overweight vs. normal group; HR_3_-adjusted hazard risk for obese vs. normal group.

A series of random-effects subgroup analyses were undertaken to examine whether the association between all-cause mortality and Asian BMI categories varied by the cohort study design (prospective vs. retrospective), geographic area, and quality of study (Fig 3A–3C).

### 1. Cohort study design

For the underweight PD patients, the pooled HR of all-cause mortality in the prospective studies was (HR 2.28, 95%CI [1.39–3.71]; p = 0.001), without heterogeneities (I^2^ = 0%; p = 0.5). The corresponding estimate for the retrospective studies was not significant (p = 0.9), with considerable heterogeneities (I^2^ = 83.1%; p = 0.02). For the overweight PD patients, the pooled HRs in both prospective[27, 30] and retrospective[28, 31] studies were not significant (p = 0.7 and 0.8) and without heterogeneities (p = 0.9 and 0.8). For the obese group, a significantly greater effect size was observed for the retrospective studies[28, 31, 32] than the prospective[27, 29, 30] studies (HR 1.90, 95%CI [1.37–2.63]; p<0.001) vs. (HR 1.16, 95%CI [1.03–1.32]; p = 0.02), with corresponding I^2^ = 4% (p = 0.4) and I^2^ = 0% (p = 0.4).

### 2. Geographic area

For the underweight PD patients, the pooled HR of all-cause mortality was significant [HR 2.28 (1.39–3.71); p = 0.001] without heterogeneities for studies conducted in non-China regions[27, 30], and was not significant (p = 0.9) with considerable heterogeneities (I^2^ = 83.1%, p = 0.02) for studies conducted in China[28, 31]. On the other hand, for the obese group, the pooled HR was significant (HR 1.60, 95%CI [1.10–2.34]; p = 0.01) for studies in China[28, 29, 31, 32] but not in non-China regions[27, 30] with substantial heterogeneities (I^2^ = 68.9%; p = 0.02). For the overweight PD patients, the pooled HRs of all-cause mortality were not significant (P = 0.8 and 0.4) for studies conducted in both China and non-China areas without heterogeneities (I^2^ = 0; p = 0.8 and I^2^ = 0; p = 0.9, respectively).

### 3. Study quality

For the underweight and overweight groups, all the studies included were ranked high quality with the same results as HR_1_ and HR_2_, respectively. For the obese group, the pooled HR for studies[27–31] with high-quality study was (1.25, 95%CI [1.03–1.52]; p = 0.02) with low heterogeneities (I^2^ = 16.1%; p = 0.3). Similar results were also found in the only fair-quality study from Zhou et al[32], (2.71, 95%CI [1.49–4.93]; p = 0.001).

### Sensitivity analysis

**Fig 4:**
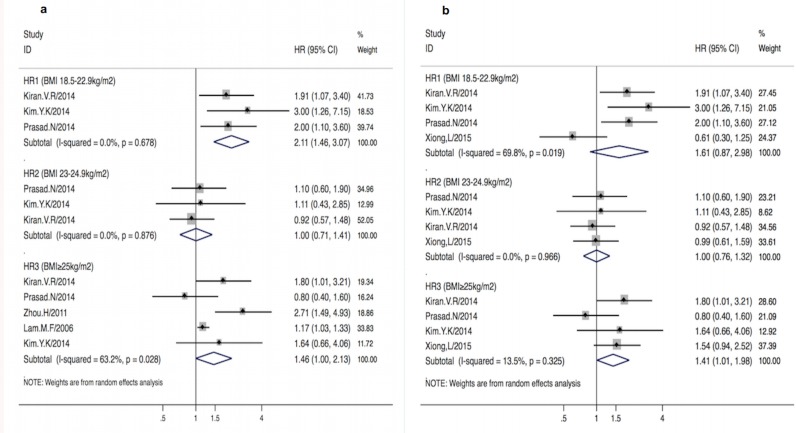
**a-b. Pooled HR**_**1-3**_ **with 95%CI of all-cause mortality in different BMI groups after excluding specific studies. *Fig 4a*** Pooled HR_1-3_ with 95%CI of all-cause mortality in different BMI groups after excluding Xiong et al’s [29] study. ***Fig 4b*** Pooled HR_1-3_ with 95%CI of all-cause mortality in different BMI groups after excluding Lam et al’ s [27] and Zhou et al’ s [30] study. Solid diamond -HR from individual study; Shadowed square behind solid diamond- statistical weight that each study contributes to the random-effect summary estimate; Hollow diamond-the overall summary HR. Horizontal line-the study specific 95%CI. HR_1-3_-adjusted hazard risk for underweight, overweight and obese group vs. normal BMI group based on Asian BMI categorization.

The influence of a single study on the overall result was estimated (Fig 4A and 4B). It was found that Xiong et al[31] had excessive influences on the summary results in the underweight group. For example, HR_1_ changed from (1.61, 95%CI [0.87–2.98]; p = 0.1) with substantial heterogeneities (I^2^ = 69.8%; p = 0.002) to (2.11, 95% [1.46–3.07]; p<0.001) without heterogeneities (I^2^ = 0%; p = 0.7). For the obese group, the strong influence on excessive heterogeneities was also observed in Lam et al[29] and Zhou et al’s[32]. After excluding these two studies, heterogeneities sharply fell from I^2^ = 57.1% (p = 0.04) to 13.5% (p = 0.3) although the effect size only diminished slightly from (1.46, 95%CI [1.07–1.98]; p = 0.02) to (1.41, 95%CI [1.01–1.98]; p = 0.045). For the overweight group, it was found that no studies asserted excessive influences on the summary HR_2_.

## Discussion

Our meta-analysis provides evidence that the relationship between BMI and all-cause mortality presents a “V-shaped curve”, indicating excessive all-cause mortality risks among the lowest (underweight) and highest (obese) Asian BMI groups, compared to the normal BMI. Besides, the obese PD patients were associated with increased CVD mortality.

In the subsequent subgroup analyses, stratified by the cohort study design, geographic area, and study quality, significant associations between the underweight group and all-cause mortality were only observed for prospective cohort study design. Studies performed in China showed a higher risk of mortality for the obese people compared to normal-weight people. No significant results were found in any subgroup analysis for the overweight group. Common explanations for these discrepancies are: (1) Typically loss of weight (a sign of malnutrition) and obese (impaired metabolic status) may lead to chronic diseases and poor prognosis, but the disease progression process is often long. As a result, prospective studies with longer follow-up and relatively larger sample size (like performed in China) tend to be more convincing; and (2) Statistically, results due to randomness were identified, due to a small number of studies included in the subgroup analyses. We tried to explore the associations between higher BMI and mortality risk in diabetic and nondiabetic PD patients, but the data were not sufficient enough for subgroup analysis (only two studies [28, 30] provided relative uncompleted data).

In the sensitivity analyses, for the association between underweight and PD mortality risk, we observed that one retrospective study[31] with younger population, reported an un-significant association, different from significant associations from other studies and common view that poor prognostic outcome in dialysis patients from malnutrition. And the pooled HR reversed to be significant with sharply falling heterogeneity, after excluding it. However, it’s reported in this study that BMI decrease more than 0.80% during the first year was an independent risk for both all-cause and CVD death. Taking these aspects into consideration, we tend to significant association between underweight and higher mortality risk among Asian PD patients.

The factors influencing BMI and the relationship between BMI and mortality risk in PD patients was well documented in previous studies, although there has been virtually no discussion devoted to the Asian PD patients. The “V-shaped” association between BMI and mortality risk among Asian PD patients could be explained with adverse effects from malnutrition and obesity. Underweight PD patients are associated with the Protein-energy Wasting (PEW) status, and consequent loss of muscle and fat mass and cachexia[35], accompanied with chronic inflammation status [36], and obesity is closely associated with a greater chance of metabolic complications due to excessive absorption of carbohydrate, high serum triglycerides, coronary calcification, catheterization failure and abdominal herniation[37–39]. Meanwhile, under the exposure of glucose load in PD solution which promotes obese, the PD patients easily gain higher BMIs. Moreover, as peritonitis is a well-known risk factor leading to increased mortality among the PD patients[40], Prasad et al [30] demonstrated that the obese PD patients had a 3.4 times greater risk of occurrence of peritonitis than that of normal BMI patients.

Unlike the plausible explanation for the “reverse epidemiology phenomenon” in HD patients, that higher BMI was associated with better survival [41], the issue of higher BMI in Asian PD patients is more complicated. Firstly, the ethnic compositions of HD cohort was different from our study which only included Asian PD patients, and Kalantar et al [42] reported that Asians were the only dialysis population in US, in whom obesity didn’t seem to confer a survival advantage. Secondly, Asian body fat percentage was under-predicted when using the equation developed in Caucasian population[43], which was also confirmed by Wang et al [44] that Asians had lower BMI but higher body fat percentage. However, the adopted BMI categorization criteria were almost for Caucasians in studies on the association between BMI and HD population. Thirdly, the BMI measure has low diagnostic accuracy in assessing body fat content, with low negative predictive value of BMI for obesity[45]. When defining obesity according to BMI, one cannot easily exclude the impact of body fluid gains, which might, influence solute clearance in PD patients, while achieving K_t_/ V_urea_ is not a big problem for HD patients. Finally, this discrepancy in mortality risks between HD and PD at higher BMI level may be attributed to different level of abdominal obesity, in the form of visceral fat mass increasing by 11–23% within the 1^st^ year of PD while visceral fat mass increase is not observed in HD patients[46]. Meanwhile, visceral fat is associated with insulin resistance, mediated by resistin secreted from adipocytes producing inflammatory markers [47], and both visceral fat and insulin resistance were found to be predictors for cardiovascular mortality in ESRD patients[48, 49].

As for the relationship between BMI and mortality in PD patients of different ethnics, higher BMI was observed to be associated with lower risk of mortality in non-Asian PD patients, such as in Brazilians[50] and Canadians[51], but not in Asian PD patients. On the contrary, similar to our conclusion, it’s been highlighted by two large-scale studies[52, 53] consisting of different ethnicities, that the relation between BMI and survival differed in Asian and non-Asian dialysis patients, and “U-shaped” association of BMI and mortality risk could be found in Asian dialysis population. For this paradoxical effect of higher BMI on PD survival, it could be speculated that obesity related nutritious protection overcomes the mortality risk from obesity among non-Asian PD patients (especially for the whites), while this association between may be reversed among Asian population.

To our best knowledge, this is the first review paper that evaluates the association of BMI with mortality among the Asian PD patients. Due to the limited pool of studies, we did not strictly set constraints in the type of PD modalities (continuous ambulatory PD, automated PD or home PD) and BMI classifications (original or modified for Asian).

Our meta-analysis has some limitations. First, BMI classification criteria were not uniform among the included studies. Some papers utilized BMI categories using the WHO recommendations for the Asian population, while others did not. Second, the analysis can be strengthened by combining some other measures such as lean body mass (LBM) [54, 55], waist circumference or waist-to-hip ratio with the BMI to induce more meaningful conclusions for the mortality risk of PD patients. For example, Ramkumar et al[56] used 24-hour urinary creatinine excretion that measures muscle mass, and concluded that the PD patients with high BMI and high or normal muscle mass had the best survival, while PD patients with high BMI but low muscle mass had the worst survival. Third, some included studies did not thoroughly adjust for essential variables such as lipoprotein (a) and CRP, which may lead to high heterogeneities. Fourth, the number of the included studies was only 7 and most of them were single-center studies with the sample size varying from 159 to 392.

## Conclusions

In conclusion, the underweight and obese Asian PD patients were found to be associated with increased all-cause mortality risk after comprehensive consideration of the results from sensitivity analyses, while the relationship between the overweight group and risk of death was not significant. More large-scale studies that include important factors would be ideal to explain the associations between BMI and mortality among the Asian PD patients.

## Supporting information

S1 Table PRISMA Checklist. (DOC)

S2 Table NEWCASTLE-OTTAWA QUALITY ASSESSMENT SCALE------- COHORT STUDIES. (DOC)

S1 File Search Strategy. (DOC)
