# Supplementary material for: The association between body mass index and mortality among Asian peritoneal dialysis patients: A meta-analysis
Source: PLoS One. 2017 Feb 16;12(2):e0172369. doi: 10.1371/journal.pone.0172369 (PMC5313204; doi:10.1371/journal.pone.0172369)
Supplement: S2 Table — (DOC) [file pone.0172369.s002.doc]

**S3 Table NEWCASTLE-OTTAWA QUALITY ASSESSMENT SCALE------- COHORT STUDIES**

| **Author’s name** | **Selection** | **Comparability** | **Outcome** | **Total score and rank** |
| --- | --- | --- | --- | --- |
|  | **(0-4*)** | **(0-2*)** | **(0-3*)** |  |
| Lam, M. F., 2006 | **＊＊＊** | **＊＊** | **＊＊** | **7 (good)** |
| Zhou, H., 2011 | **＊＊** | **＊＊** | **＊** | **5 (fair)** |
| Unal, A. 2013 | **＊＊＊** | **＊** | **＊** | **5 (fair)** |
| Kim, Y. K. 2014 | **＊＊＊＊** | **＊＊** | **＊＊** | **8 (good)** |
| Kiran,V.R 2014 | **＊＊＊** | **＊＊** | **＊＊** | **7 (good)** |
| Prasad.N 2014 | **＊＊＊** | **＊＊** | **＊＊** | **7 (good)** |
| Xiong,L 2015 | **＊＊＊** | **＊＊** | **＊＊＊** | **8 (good)** |
