# Supplementary material for: The association between body mass index and mortality among Asian peritoneal dialysis patients: A meta-analysis
Source: PLoS One. 2017 Feb 16;12(2):e0172369. doi: 10.1371/journal.pone.0172369 (PMC5313204; doi:10.1371/journal.pone.0172369)
Supplement: S1 File — (DOC) [file pone.0172369.s003.doc]

**S2 File Search Strategy**

**Search Strategy:**

**Database1: Pubmed**

#1 Body Mass Index [MESH Terms]

#2 Obesity [MESH Terms]

#3 Overweight [MESH Terms]

#4 Peritoneal dialysis [MESH Terms]

#5 Continuous Ambulatory Peritoneal Dialysis [MESH Terms]

#6 Mortality [All fields]

#7 Mortality risk [All fields]

#8 Survival [All fields]

#9 #1 or #2 or #3

#10 #4 or #5

#11 #6 or #7 or #8

#12 #9 and #10 and #11

#13 #12 Fliters: English

**Database 2: Embase <1974 to 2016 Week 19>**

#1 exp continuous ambulatory peritoneal dialysis/

#2 exp peritoneal dialysis/

#3 exp body mass/

#4 exp obesity/

#5 exp overweight/

#6 exp mortality/

#7 mortality risk.mp.

#8 exp survival/

#9 #1 or #2

#10 #3 or #4 or #5

#11 #6 or #7 or # 8

#12 #9 and #10 and #11

#13 limit #12 to English language
